# The prognostic potential of RNA in stage II colon cancer: Insights from a screened multicenter population‐based cohort study

**DOI:** 10.1002/ijc.35507

**Published:** 2025-06-06

**Authors:** Ulrik Korsgaard, Maria P. Kristensen, Juan L. García‐Rodríguez, Sanne Kjær‐Frifeldt, Jan Lindebjerg, Torben F. Hansen, Jørgen Kjems, Henrik Hager, Lasse S. Kristensen

**Affiliations:** ^1^ Department of Clinical Pathology Lillebaelt Hospital, University Hospital of Southern Denmark Vejle Denmark; ^2^ Danish Colorectal Cancer Center South Lillebaelt Hospital, University Hospital of Southern Denmark Vejle Denmark; ^3^ Institute of Regional Health Research University of Southern Denmark Odense Denmark; ^4^ Department of Biomedicine Aarhus University Aarhus Denmark; ^5^ Department of Oncology Lillebaelt Hospital, University Hospital of Southern Denmark Vejle Denmark; ^6^ Department of Molecular Biology and Genetics Aarhus University Aarhus Denmark; ^7^ Interdisciplinary Nanoscience Center (iNANO) Aarhus University Aarhus Denmark; ^8^ Department of Pathology Aarhus University Hospital Aarhus Denmark

## Abstract

The management of stage II colon cancer poses an important clinical challenge due to difficulties in identifying patients at risk of recurrence. RNA expression profiles in solid tissues have demonstrated a strong potential as prognostic markers. The objective of this study was to develop an RNA‐based risk score to risk‐stratify patients with stage II colon cancer using a large, well‐characterized, and screened patient cohort. This multicenter cohort study encompasses tissue samples collected from all surgically resected stage II colon cancer patients in the Region of Southern Denmark from 2014 to 2016 (*n* = 739). Gene expression analysis was performed on 122 RNAs, previously described as holding prognostic potential in colorectal cancer, using NanoString nCounter analyses. The resulting RNA expression profiles were used to develop a score predicting the risk of recurrence. The primary endpoint for the study was time to recurrence (TTR), with overall survival (OS) as a secondary endpoint. A dichotomized score, derived from the combined expression of four RNAs (*ZNF697*, *SNORA2B*, *CTSC* and *OXLD1*), effectively predicted TTR in stage II colon cancer patients. The score revealed a hazard ratio (HR) of 6.84 in univariate analysis (*p* < 0.001) and a HR of 5.00 in multivariate analysis (p < 0.001), surpassing the prognostic performance of known clinical risk factors. Additionally, the score was significantly associated with OS in Kaplan–Meier analysis (*p* = 0.024). In conclusion, this four‐gene expression score demonstrates a strong association with TTR in stage II colon cancer patients, providing valuable prognostic insights that extend beyond conventional clinical risk markers.

## What's New?

Patients with stage II colon cancer exhibit considerable variation in prognosis. Consequently, decisions regarding the necessity of adjuvant chemotherapy in this population often rely on imperfect high‐risk criteria. Here, the authors developed a novel RNA‐based risk score, using NanoString nCounter technology, to stratify recurrence risk among stage II colon cancer patients. This score, which incorporates four RNAs—*ZNF697*, *CTSC*, *SNORA2B*, and *OXLD1*—demonstrates significant predictive power for time to recurrence and overall survival, outperforming traditional clinical risk factors. The findings underscore the potential of RNA expression profiles as biomarkers for enhancing personalized treatment strategies in stage II colon cancer.

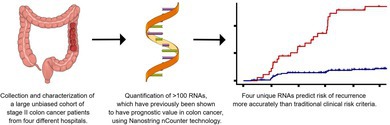

**Abbreviations**
AUC: area under the curve
CI: confidence interval
circRNA: circular RNA
DCCG: Danish Colorectal Cancer Group
dMMR: mismatch repair deficient
HR: Hazard ratio
IQR: Inter quartile range
lncRNA: long non‐coding RNA
mRNA: messenger RNA
OR: odds ratio
pMMR: mismatch repair proficient
RNA‐seq: RNA sequencing
ROC: receiver operating characteristics
rRNA: ribosomal RNA
RT‐qPCR: reverse transcription‐quantitative polymerase chain reaction
snoRNA: small nucleolar RNA
TCGA: The Cancer Genome Atlas
TTR: time to recurrence
vtRNA: vault RNA

## 1 INTRODUCTION

Patients with stage II colon cancer constitute a heterogeneous group with a variable prognosis, and determining the necessity of adjuvant chemotherapy treatment remains a clinical challenge.^1^, ^2^ Currently, patients with stage II colon cancer are generally only considered for adjuvant chemotherapy treatment if they have a mismatch repair proficient (pMMR) tumor, as patients with a mismatch repair deficient tumor (dMMR) do not benefit from adjuvant chemotherapy as monotherapy.^3^, ^4^ Patients with pMMR tumors are potential candidates for adjuvant chemotherapy if they have certain high‐risk criteria. Although high‐risk criteria vary between countries, they generally include acute surgery, anastomosis leakage, lymphatic invasion, venous invasion, perineural invasion, <12 lymph nodes sampled, pT4‐stage, and signet ring cell carcinoma.^5^, ^6^, ^7^ Despite these stringent criteria, it is still questioned whether using them for treatment decisions improves patient outcomes.^8^, ^9^, ^10^

Advances in total RNA sequencing (RNA‐seq) have facilitated the discovery of many different classes of RNAs with potential oncogenic or tumor suppressor functions, including messenger RNAs (mRNAs), long non‐coding RNAs (lncRNAs), circular RNAs (circRNAs), small nucleolar RNAs (snoRNAs), and vault RNAs (vtRNAs). circRNA is a relatively newly discovered class of RNA with exceptional stability^11^, ^12^ and important roles in cancer.^13^ snoRNAs play an important function in the chemical modification of ribosomal RNA (rRNA) and other types of RNAs, such as spliceosomal RNAs,^14^ and promote tumorigenesis in colon cancer.^15^, ^16^ The role of vtRNAs in cancer progression is not fully understood, but they may have the potential to hinder apoptosis^17^ and increase proliferation.^18^

Aside from having oncogenic or tumor‐suppressing functions, many types of RNAs have a strong biomarker potential in colon cancer. Often, a combination of the expression levels of several RNAs in a tumor is used to generate a signature that can predict patient outcome. A well‐described RNA signature in colon cancer is the Oncotype DX Colon Recurrence Score®, which is based on mRNA quantification with RT‐qPCR.^19^ This signature has subsequently been validated in multiple studies, showing that it could predict the risk of relapse in stage II colon cancer.^20^, ^21^, ^22^ Another well‐described and independently validated gene signature is the mRNA‐based ColoPrint signature.^23^, ^24^ Several other studies have developed scores based on microarray or total RNA‐seq data for mRNAs,^25^, ^26^ lncRNAs^27^, ^28^ and circRNAs.^29^ However, none of these scores has undergone validation in independent studies.

Importantly, the most thorough previous biomarker studies with RNA signatures based on solid tissue were conducted on cohorts from the 1980's to early 2000's.^20^, ^21^, ^22^, ^24^ Since then, the implementation of colon cancer screening in many Western countries has resulted in colon cancer being detected at earlier stages.^30^ Furthermore, modern preoperative CT‐scans and extensive operative lymph node sampling reduce the risk of undiagnosed stage III or IV tumors being present in a modern stage II colon cancer cohort. As such, there is a pressing need for biomarker investigations in the context of colon cancer screening, as well as modern surgical, radiologic and oncologic treatment regimes.

In this study, our aim was to re‐evaluate some of the most promising RNA transcripts described in the literature^19^, ^23^, ^25^, ^27^, ^28^, ^29^, ^31^ using an unbiased cohort sampled in a screened population. Moreover, we aimed to develop a gene signature based on a clinically applicable method with the ability to identify high‐risk patients with stage II colon cancer within a large and extensively characterized cohort. To achieve this, we designed a custom NanoString nCounter® panel encompassing the most promising mRNAs, lncRNAs, circRNAs, snoRNAs, and vtRNAs from the literature. We then assessed their potential as prognostic biomarkers, with a specific focus on predicting the risk of recurrence within 5 years following primary diagnosis.

## 2 MATERIALS AND METHODS

### 2.1 Patient cohort

The study is designed as a multicenter retrospective cohort study, and the cohort has been described previously.^32^ In brief, the patient cohort consists of all patients resected for UICC stage II colon cancer in the Region of Southern Denmark between 2014 and 2016. Patients were identified using the Danish Colorectal Cancer Group (DCCG) database and the Danish Pathology System (*N* = 739). Patients were from four different hospitals (Vejle Hospital, Odense Hospital, Aabenraa Hospital and Esbjerg Hospital). After collection of the tissue specimens from the pathology archives, all histological tumor slides were reevaluated and restaged according to the latest UICC classification (8th edition) and were reassessed for the following histologic findings: pT‐category, histological subtype (including mucinous and regular adenocarcinomas, as shown in Table 1), tumor differentiation, lymphatic invasion, venous invasion, and perineural invasion. Tumor localization was also recorded, including anatomical sites, as outlined in Table 1.

**TABLE 1:** Patient characteristics.

Variable	All patients	No recurrence	Recurrence
	*N* = 471^a^	*N* = 432^a^	*N* = 39^a^
Age	73 (67, 80)	73 (67, 80)	74 (69, 79)
Sex			
Female	252 (54)	234 (54)	18 (46)
Male	219 (46)	198 (46)	21 (54)
Histological Subtype			
Adenocarcinoma	404 (85.8)	372 (86)	32 (82)
Mucinous adenocarcinoma	63 (13.4)	56 (13)	7 (18)
Medullary carcinoma	3 (0.6)	3 (0.7)	0 (0)
Undifferentiated carcinoma	1 (0.2)	1 (0.2)	0 (0)
Tumor differentiation			
Moderate	433 (92)	396 (92)	37 (95)
Poor	38 (8)	36 (8)	2 (5)
Tumor location			
Cecum	79 (16.8)	77 (18)	2 (5.1)
Ascending Colon	83 (17.6)	79 (18)	4 (10)
Right Colic Flexure	36 (7.6)	36 (8.3)	0 (0)
Transverse Colon	43 (9.1)	40 (9.3)	3 (7.7)
Left Colic Flexure	31 (6.6)	25 (5.8)	6 (15)
Descending Colon	34 (7.2)	32 (7.4)	2 (5.1)
Sigmoid Colon	165 (35)	143 (33)	22 (56)
Mismatch Repair Status			
dMMR	117 (25)	112 (26)	5 (13)
pMMR	354 (75)	320 (74)	34 (87)
pT‐category			
pT3	420 (89)	393 (91)	27 (67)
pT4	51 (11)	39 (9)	12 (31)
Lymphnodes			
<12	9 (2)	7 (2)	2 (5)
>12	462 (98)	425 (98)	37 (95)
Acute intervention			
Yes	46 (10)	35 (8)	11 (28)
No	425 (90)	397 (92)	28 (72)
Anastomosis leakage			
Yes	15 (3)	13 (3)	2 (5)
No	456 (97)	419 (97)	37 (95)
Venous invasion			
Yes	145 (31)	131 (30)	14 (36)
No	326 (69)	301 (70)	25 (64)
Lymphatic invasion			
Yes	25 (5)	23 (5)	2 (5)
No	446 (95)	409 (95)	37 (95)
Perineural invasion			
Yes	59 (13)	49 (11)	10 (26)
No	412 (87)	383 (89)	29 (74)
Received adjuvant chemotherapy			
Yes	63 (13)	52 (12)	11 (28)
No	408 (87)	380 (88)	28 (72)
Follow up (months)	87 (72, 99)	88 (77, 100)	57 (29, 80)
Time to recurrence (months)	–	–	21 (13, 37)
Death			
Yes	88 (19)	65 (15)	23 (59)
No	384 (81)	367 (85)	16 (41)
Overall survival (months)	87 (72, 99)	88 (76, 100)	57 (29, 80)

^a^ Median (IQR); *n* (%).

**FIGURE 1:**
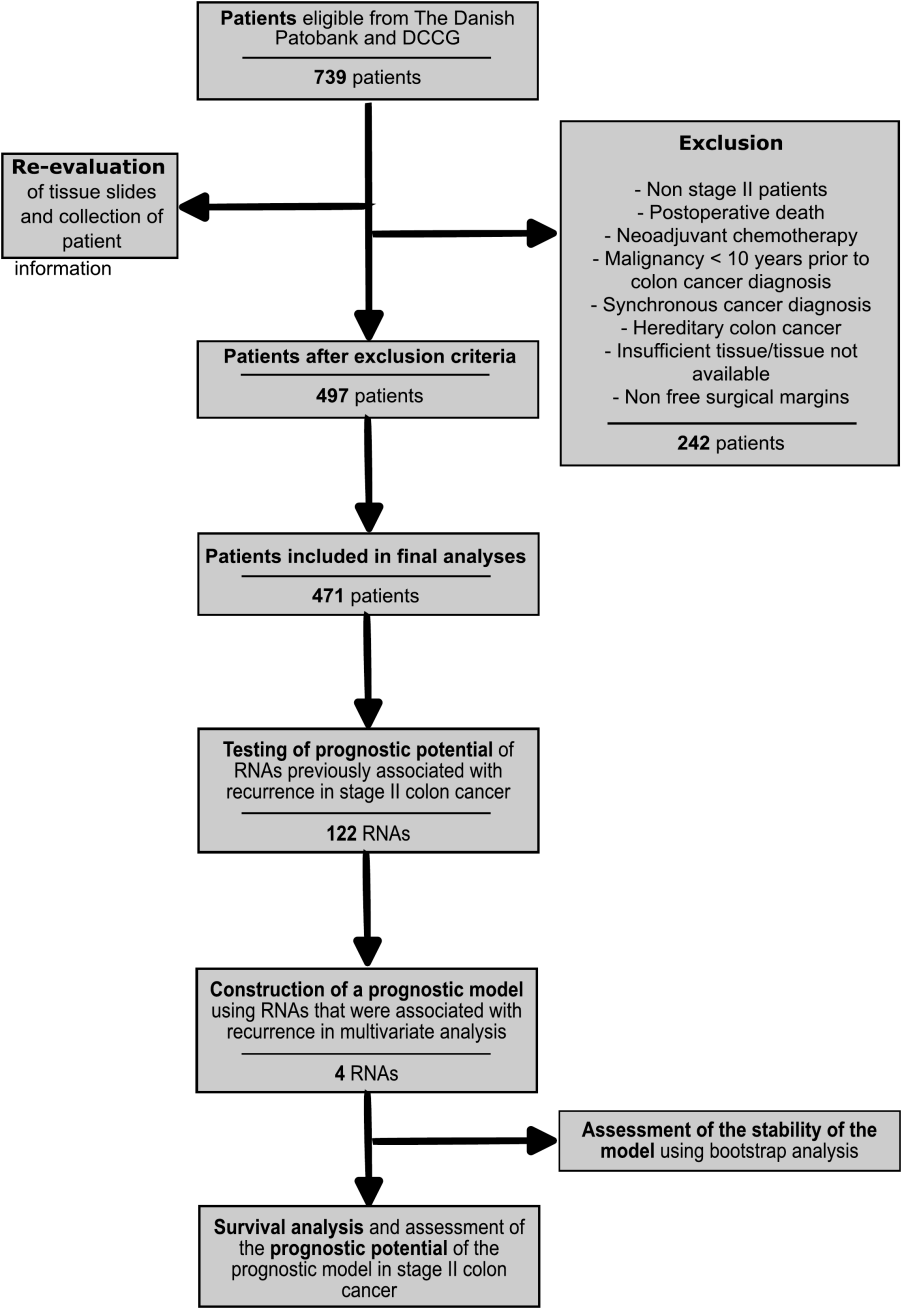
Schematic overview of the workflow and patient inclusion and exclusion. In total, 739 patients were detected through the Danish Colorectal Cancer Groups (DCCGs) database; 242 patients were excluded based on the exclusion criteria listed. After exclusion criteria, 497 patients remained, of which 26 were excluded because of non‐satisfactory nCounter® results. In total, 471 patients constituted the final study population.

Patients were included in the study according to the following criteria: histologically verified colon adenocarcinoma UICC stage II, complete resection (*R*_0_) and age ≥18 years. Patients were excluded if they met one of the following criteria: received neoadjuvant chemotherapy, had synchronous tumors (defined as another tumor diagnosed <4 months after primary colon cancer diagnosis), another malignant disease up to 10 years prior to primary colon cancer (non‐melanoma skin‐cancer excepted), post‐operative death within 3 months, or hereditary cancer (familial adenomatous polyposis or Lynch syndrome). In total, 242 patients were excluded, and the final study population comprised 497 patients (Figure 1). All patients were treated according to the DCCG guidelines.^5^, ^33^ MMR status was assessed with immunohistochemistry, and subsequent analysis of methylation status of promoter regions and genetic analyses were carried out if necessary to rule out hereditary causes of colon cancer. Patients received standard of care follow‐up, which includes postoperative CT scans at 12 and 36 months and colonoscopies at 3 and 60 months after surgery. All the clinical patient data were collected manually from the electronic patient records.

### 2.2 Tumor tissue

All tumor tissue was collected during routine pathology diagnostics and fixed in 10% neutral‐buffered formalin for at least 48 h before dehydration and paraffin embedding. After diagnostic tests, the tissue was stored at the respective pathology departments in a cool, ventilated environment. All tissue blocks were retrieved from the pathology archives, and the tumor slide representing the deepest invasion of the tumor was selected for further investigation.

### 2.3 RNA extraction

Two consecutive sections of 15 μm were cut from each of the paraffin‐embedded samples, and RNA was purified with the Maxwell® CSC RNA FFPE Kit (Promega, Madison, WI) according to the manufacturer's protocol. The RNA yield was measured with the NanoDrop™ One/One^C^ Microvolume UV–Vis Spectrophotometer (Thermo Fisher Scientific, Waltham, MA).

### 2.4 NanoString nCounter® analysis

We designed a custom NanoString nCounter® code set (NanoString Technologies, Seattle, WA) for mRNAs, lncRNAs, and circRNAs from signatures with prognostic potential in stage II colon cancer.^19^, ^23^, ^25^, ^27^, ^28^, ^29^ Additionally, we included mRNAs, lncRNAs, circRNAs, snoRNAs, and vtRNAs that demonstrated prognostic potential in our previous RNA‐seq study.^31^ For normalization purposes, we included nine reference genes: *GPX1*, *PUM1*, *PGK1*, *UBB*, *SF3A1*, *ATP5F1E*, *VDAC2*, *ACTB*, and *MRPL19*. Furthermore, we included the tissue specific markers *ACTA2* (smooth muscle), *DES* (smooth muscle), *VIM* (stromal cells), *CALB2* (nerve tissue), *CDX2* (colon epithelial cells), *SATB2* (colon epithelial cells) and *KRT8* (epithelial cell marker). The final panel included a total of 158 different target transcripts (Table S1).

NanoString nCounter® analyses were performed according to the manufacturer's protocol using a total input of 100 ng RNA from each sample. Samples from patients where more than 50 targets had raw read counts below background levels, defined as the mean of negative controls plus two standard deviations, were removed from further analyses (*n* = 26) (Figure 1). The data were normalized to the mean of the two reference genes with the lowest coefficient of variation (CV), *GPX1* and *PUM1*, using the nSolver 4.0 software. Targets detected in fewer than 39 samples were excluded from further analyses to ensure the potential detection of RNAs exclusively expressed in patients with recurrence. The final number of targets, excluding housekeeping genes, tissue specific markers, and undetected targets, used in downstream analyses was 122 (marked in bold in Table S1).

### 2.5 Statistical analyses

All statistical analyses were performed in R‐studio (version 4.2.2). Receiver operating characteristics (ROC) analyses were conducted with the R package caTools (v. 1.18.2). Univariate and multivariate logistic regression was conducted with 5‐year recurrence as the outcome using the R package, stats (v. 3.6.2). For logistic regression analyses, results are reported as odds ratios (ORs), confidence intervals (CIs) and *p*‐values. RNA transcripts that were significant in univariate analysis with the highest area under the curve (AUC) measured through ROC analysis were subjected to a multivariate logistic regression analysis. Differentially expressed RNAs, which were significant in multivariate analysis, were combined in a final panel consisting of four transcripts, and the combined 5‐year prognostic potential was measured through ROC analysis. Bootstrap analysis was conducted with 10,000 iterations to assess the stability of the final model, and the mean AUC and the bias‐corrected and accelerated bootstrap (BC_a_) CIs were reported. The regression coefficients of the final model were used to calculate a risk score, and patients were dichotomized according to the Youden index^34^ into a low‐risk and a high‐risk group. The primary endpoint assessed for the classical clinicopathological criteria and the risk score was time to recurrence (TTR) with overall survival (OS) as a secondary endpoint. TTR was defined as the time from surgery to relapse or death from colon cancer, and patients were censored if they died of other causes. OS was defined as death from any cause. Data regarding OS were available for all patients. Results of cox proportional hazard analysis are reported as hazard ratios (HRs), CIs, and *p*‐values. Kaplan–Meier analysis was used to visualize the risk score's association with TTR and OS. In univariate and multivariate cox proportional hazard analysis, clinicopathological criteria known to be associated with the risk of recurrence were assessed. The clinicopathological criteria included pT‐category, mismatch repair status, number of lymph nodes assessed, acute surgical intervention, anastomosis leak, venous invasion, lymphatic invasion, and perineural invasion.

## 3 RESULTS

### 3.1 Patient characteristics

After exclusion of patients providing insufficient NanoString nCounter® results, as described in the methods section, 471 patients remained in the final cohort. Among these, 39 (8.3%) experienced relapse within 5 years. There was no difference in age, sex, histological subtype, tumor differentiation, MMR‐status, number of lymph nodes assessed, anastomosis leakage, venous invasion, or lymphatic invasion between patients with and without recurrence. Patients with recurrence had a higher incidence of tumors in the left colic flexure and sigmoid colon, were more likely to have a pT4 tumor, receive adjuvant chemotherapy, undergo acute surgery, and exhibit perineural invasion. The median follow‐up time for the total cohort was 87 months (IQR = 72, 99). The median TTR was 21 months (IQR = 13, 37). Of the 39 patients who suffered from relapse within 5 years, 23 (59%) died, while 65 (15%) of the 432 patients without recurrence died (Table 1).

### 3.2 No difference in tissue composition between patients with and without recurrence

First, to avoid potential confounding due to cell‐type specific expression,^35^ we evaluated the expression levels of the tissue‐specific mRNAs included in the nCounter® panel (*DES, ACTA2*, *VIM*, *CALB2*, *CDX2*, *SATB2* and *KRT8*) to investigate whether the tissue composition between patients with and without recurrence was consistent. This analysis revealed no statistically significant differences in tissue specific markers between patients with recurrence and those without (Figure S1).

### 3.3 Development of a prognostic RNA panel

Next, RNA transcripts from previously described promising RNA signatures^19^, ^23^, ^25^, ^27^, ^28^, ^29^ were evaluated individually in the context of our screened stage II colon cancer cohort. Specifically, the prognostic ability of the RNAs to predict the 5‐year risk of recurrence was assessed through univariate logistic regression. The analysis showed that 12 RNAs were significantly associated with recurrence (*p* < 0.05) (Table S2). Similarly, the 58 RNA transcripts from our previous RNA‐seq study^31^ were analyzed. The analysis revealed eight RNAs significantly associated with recurrence (*p* < 0.05) (Table S3).

Next, to mitigate the risk of overfitting the logistic regression model, we performed a feature selection process based on the univariate analyses. Specifically, we selected the 10 RNA transcripts that were significant in univariate analysis with the strongest association to 5‐year risk of recurrence in ROC analyses. Among the RNAs with the strongest prognostic potential, *ZNF697, CDC6, IL2RB, MKI67*, and *CTSC* were from previously described RNA signatures and had AUCs of 0.675, 0.634, 0.632, and 0.619, respectively. *MAPRE3, SNORA2B, IFI35, OXLD1*, and *SNHG1* were from our previous RNA‐seq study and had AUCs of 0.638, 0.601, 0.612, 0.618, and 0.609, respectively (Table S4). In multivariate analysis, *ZNF697*, *OXLD1*, *SNORA2B*, and *CTSC* remained significant (Table 2).

**TABLE 2:** Multivariate logistic regression analysis of RNA transcripts association with 5‐year recurrence.

RNA	OR	95% CI	*p* value
*ZNF697*	2.23	1.22–4.20	0.011
*MAPRE3*	1.56	0.82–2.99	0.176
*CDC6*	1.57	0.78–3.30	0.218
*IL2RB*	0.94	0.52–1.69	0.839
*MKI67*	0.65	0.30–1.33	0.242
*SNORA2B*	2.48	1.19–5.41	0.019
*IFI35*	0.82	0.35–1.88	0.647
*CTSC*	0.30	0.11–0.84	0.021
*OXLD1*	0.21	0.07–0.59	0.004
*SNHG1*	0.76	0.26–2.15	0.611

Since we observed a significant association between *ZNF697*, *SNORA2B*, *CTSC*, *OXLD1* and the 5‐year risk of recurrence in multivariate logistic regression analysis, we assessed the collective predictive performance of these four RNAs. Consequently, a risk score was calculated for each patient based on the expression of the RNA and the coefficients of the four transcripts in multivariate logistic regression with the following formula:

$$\text{logit}\left(\text{risk}-\text{score}\right)=0.456+0.988\times {\mathrm{Exp}}_{\mathrm{ZNF}697}+1.036\times {\mathrm{Exp}}_{\text{SNORA}2B}–1.308\times {\mathrm{Exp}}_{\text{CTSC}}–1.556\times {\mathrm{Exp}}_{\text{OXLD1}}$$

**FIGURE 2:**
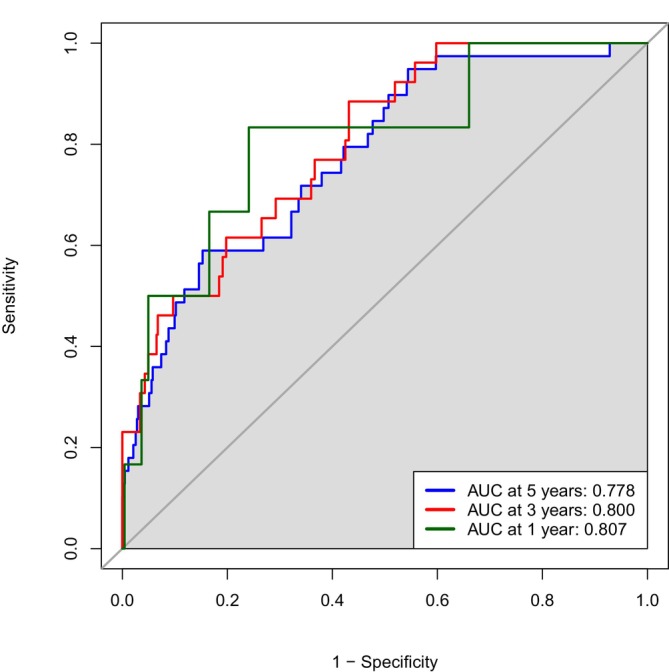
Receiver operating characteristics. The four RNAs were combined in multivariate analysis and assessed collectively as a prognostic marker for 1‐year, 3‐year, and 5‐year risk of recurrence. Based on the coefficients of the four RNAs in multivariate logistic regression, for 5‐year risk of recurrence, a risk score was calculated. Youden's index (0.131) was used to divide patients into a high‐risk and low‐risk group.

As anticipated, the combined risk‐score exhibited superior predictive capabilities compared to the individual RNAs. In ROC analysis, the risk‐score yielded an AUC of 0.778 at 5 years, while the AUC was 0.800 and 0.807 for 3 years and 1 year, respectively (Figure 2). The stability of the model was assessed through bootstrap analysis, which provided a mean bootstrap AUC of 0.776 (95% CI, 0.694–0.845) (Figure S2).

### 3.4 A dichotomized risk score is significantly associated with TTR and OS

In a clinical setting, dichotomized variables are often preferred as they are easier to interpret. Hence, the score was divided into a high‐risk and a low‐risk group based on the optimal cutoff (Youden index) from the ROC analysis (Figure 2). The high‐risk group comprised 89 patients, among whom 23 (25.8%) experienced recurrence, while the low‐risk group comprised 382 patients, of which 16 (4.2%) experienced recurrence. To assess whether the high‐risk and low‐risk groups had any association with clinical markers known to have prognostic significance, an assessment of the risk‐score's association with clinicopathological data was conducted. No significant differences in age, sex, histological subtype, tumor differentiation, number of lymph nodes assessed, presence of anastomosis leak or lymphatic invasion were noted. However, a high‐risk score was significantly associated with venous invasion, pT4 stage, left‐sided tumors, acute intervention, pMMR, and perineural invasion (Table S5).

The association between the risk‐score as well as the clinicopathological criteria with TTR was evaluated through both univariate and multivariate cox regression analysis. In the univariate analysis, a high risk‐score, acute intervention, pT4‐category, and pMMR were significantly associated with TTR. In multivariate analysis, only a high‐risk score and pT4‐category remained significant (Table 3).

**TABLE 3:** Association of the risk‐score and high‐risk criteria with time to recurrence in univariate and multivariate cox regression.

Variable	Univariate			Multivariate		
	HR	95% CI	*p*‐value	HR	95% CI	*p*‐value
Risk‐score, high vs. low	6.84	3.61–12.95	<0.001	5.00	2.52–9.93	<0.001
Acute intervention, yes vs. no	4.15	2.06–8.34	<0.001	1.73	0.77–3.92	0.19
pT‐category, pT4 vs. pT3	3.96	2.01–7.82	<0.001	2.56	1.21–5.42	0.01
Perineural invasion, yes vs. no	2.61	1.27–5.36	0.016	1.67	0.79–3.50	0.18
Mismatch Repair Status, pMMR vs. dMMR	2.33	0.91–5.96	0.077	1.74	0.66–4.60	0.27
Lymph nodes, <12 vs. >12	2.71	0.65–11.25	0.169	2.59	0.61–10.96	0.20
Anastomosis leak, yes vs. no	1.91	0.46–7.94	0.371	1.63	0.38–6.99	0.51
Venous invasion, yes vs. no	1.27	0.66–2.44	0.474	0.94	0.48–1.84	0.86
Lymphatic invasion, yes vs. no	0.95	0.23–3.93	0.941	1.01	0.23–4.38	0.99

**FIGURE 3:**
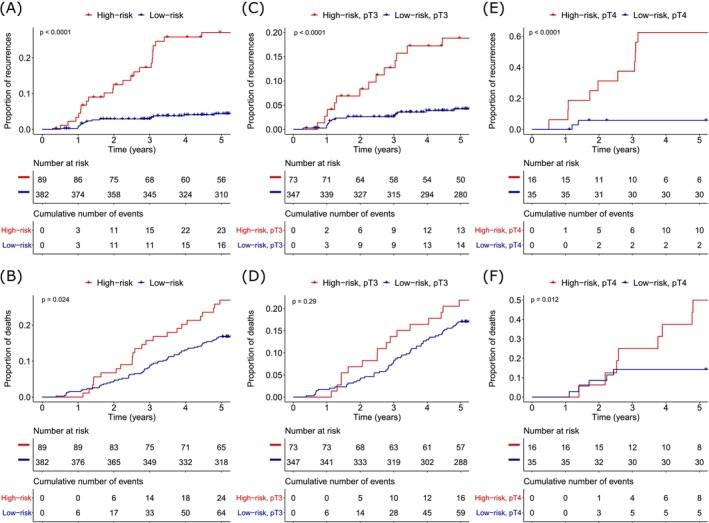
Kaplan–Meier Analyses. (A) The risk‐score's association with time to recurrence (TTR) (in years) after surgery for high‐risk patients (*n* = 89) and low‐risk patients (*n* = 382). (B) The risk‐score's association with overall survival (OS) (in years) after surgery for high‐risk patients (*n* = 89) and low‐risk patients (*n* = 382). (C) The risk‐score's association with TTR (in years) after surgery for high‐risk, pT3 patients (*n* = 73) and low‐risk, pT3 patients (*n* = 347). (D) The risk‐score's association with OS for high‐risk, pT3 patients (*n* = 73) and low‐risk, pT3 patients (*n* = 347). (E) The risk‐score's association with TTR for high‐risk, pT4 patients (*n* = 16) and low‐risk, pT4 patients (*n* = 35). (F) The risk‐score's association with OS for high‐risk, pT4 patients (*n* = 16) and low‐risk, pT4 patients (*n* = 35).

In Kaplan–Meier analysis, the risk‐score was significantly associated with shorter TTR (Figure 3A) and lower OS, as determined by the log‐rank test (Figure 3B). When stratifying high‐risk and low‐risk patients according to chemotherapy treatment, it was revealed that high‐risk patients who received adjuvant chemotherapy had a worse TTR compared to high‐risk patients who did not receive chemotherapy (Figure S3A). Patients with a low‐risk score who received adjuvant chemotherapy treatment showed the same TTR as low‐risk patients who did not receive chemotherapy (Figure S3B). When dividing patients according to the median of the risk‐score, the high‐risk group encompassed 32 out of 39 recurrences (Figure S3B). Since multivariate cox regression analysis revealed that pT‐category and the risk‐score were significantly associated with TTR, separate Kaplan–Meier analyses were conducted for pT3‐category and pT4‐category patients. For pT3 patients, the score effectively identified patients with a shorter TTR (Figure 3C), although no significant association with OS was observed (Figure 3D). However, it became evident that the risk‐score excelled in identifying patients with a short TTR among those with pT4 tumors, as the high‐risk group encompassed 10 out of 12 patients with recurrence (Figure 3E). Furthermore, patients with a pT4 tumor and a high‐risk score had a significantly shorter OS than patients with a pT4 tumor and a low‐risk score (Figure 3F).

## 4 DISCUSSION

Stage II colon cancer often has a favorable prognosis but presents a considerable challenge in clinical oncology, as identifying patients who benefit from adjuvant chemotherapy remains difficult, and studies using traditional clinical markers for risk stratification yield contradicting results.^8^, ^10^ Although blood‐based circulating tumor DNA has shown promise as a prognostic biomarker in recent years, its sensitivity may not always be adequate for the detection of minimal residual disease.^36^ Therefore, biomarkers based on solid tissue are still relevant in this setting, and the identification of new reliable prognostic solid tissue specific markers that can stratify patients based on their risk of recurrence is of paramount importance. Here, we re‐evaluated the most promising RNA transcripts described in the literature,^19^, ^23^, ^25^, ^27^, ^28^, ^29^ including mRNAs, lncRNAs, circRNAs, snoRNAs, and vtRNAs, using a reproducible and clinically applicable method known as NanoString nCounter®.^37^ We used an unbiased cohort of patients from four different hospitals in Denmark to ensure that the results are applicable to the broader population. From these data, we developed a risk score based on the expression of four RNA transcripts, which consists of three mRNAs (*ZNF697*, *OXLD1* and *CTSC*) and one snoRNA (*SNORA2B*). The risk score predicts TTR more accurately than traditional clinical markers and was significantly associated with OS.

The NanoString nCounter® methodology was chosen for quantification of gene expression for several reasons. Primarily, it functions without enzymes that may introduce bias and artifacts during cDNA synthesis and amplification, as seen in methods such as qPCR and RNA‐seq. Especially for circRNAs, quantification through qPCR can pose a significant problem, as rolling circle amplification can lead to overestimation of the abundance of circRNAs.^38^, ^39^ Moreover, the simple workflow and few hands‐on steps render it more reproducible. The method also excels in its applicability to FFPE tissues, in contrast to RNA‐seq, which heavily relies on non‐degraded RNA from fresh frozen tissues. Furthermore, the method is already employed at many pathology departments where it is used to assess the gene signature, PAM50,^40^, ^41^ which is used to subset breast cancer patients that do not benefit from adjuvant chemotherapy. Thus, the risk‐score developed in this study could be seamlessly integrated into future pathology reports. In contrast, earlier investigations addressing the same clinical issue often utilized fresh frozen tissues and employed techniques such as qPCR, RNA‐seq, or microarrays, either individually or in combination.^19^, ^23^, ^25^, ^27^, ^28^, ^29^

In the present study, we included RNA transcripts that exhibited promise as prognostic markers in previous research.^19^, ^23^, ^25^, ^27^, ^28^, ^29^ In contrast to previous studies, our study utilized a meticulously characterized cohort with complete data for all known clinical risk markers. In addition, the cohort was collected in a population invited to participate in the national colon cancer screening program in Denmark, thus making it representative of future patient demographics. Moreover, the absence of selection bias in patient recruitment was ensured by Denmark's unique personal identification number system, enabling the identification of all patients diagnosed with stage II colon cancer in the entire Region of Southern Denmark, which represents approximately 20% of the entire population of Denmark. In contrast, previous studies relied on cohorts assembled for various purposes, where selection bias is likely to be introduced.^19^, ^23^, ^25^, ^27^, ^28^, ^29^ Another unique feature of our unbiased cohort is that it solely consists of patients with stage II colon cancer, in contrast to other studies having used a mix of stage II, stage III cancers and in some cases stage IV cancers.^19^, ^23^, ^25^, ^27^, ^28^, ^29^ Two of the RNA signatures from previous studies were subsequently validated in external cohorts of stage II colon cancers, where they also provided significant prognostic information. However, these validation studies^20^, ^21^, ^22^, ^24^ utilized tissues from patients gathered before colon cancer screening programs were initialized with cohorts assembled between 1987 and 2003. In addition to colon cancer screening, advances in surgical procedures, radiologic modalities, oncologic treatment, and histopathologic techniques must be expected to have considerably improved the outcomes of patients and thus decrease the risk of recurrence and overall survival of patients with stage II colon cancer today.^42^ Hence, the previous studies reported higher recurrence rates ranging from 15% to 25%, while the patient cohort in the present study had a relatively low recurrence rate of 8.3%, reflecting what other recent studies have reported.^42^, ^43^, ^44^

Many of the RNA transcripts from the previously reported RNA signatures may have failed in our univariate logistic regression analysis due to the dissimilarities in cohort design and gene quantification methods. We chose not to validate gene signatures as a whole because of the different methods used for quantification and the unavailability of probes for all target genes or probes failing to meet our detection cutoffs.

Instead, a panel was created by combining the four most promising candidates based on their ability to predict the risk of recurrence within 5 years in multivariate analysis. This panel included two genes from a former RNA signature (*ZNF697* and *CTSC*)^23^ and two from our previous RNA‐seq study.^31^ This panel successfully stratified patients into distinct high‐risk and low‐risk groups. Notably, the high‐risk group exhibited a significantly shorter TTR, with a HR of 6.84 compared to the low‐risk group in univariate analysis. The patients within the high‐risk group more often had pT4 tumors and perineural invasion, indicating that high‐risk patients may have a more aggressive disease phenotype. However, in multivariate analysis, the risk score emerged as a robust predictor of TTR, surpassing the predictive capacity of other well‐established risk criteria, including that of pT‐category and perineural invasion. In multivariate analysis, pT‐category was shown to have a prognostic impact beyond that of the risk score alone. Hence, the inclusion of pT‐category in a final predictive model seemed logical, especially considering the established significance of pT‐category stage II colon cancer as a prognostic marker^44^ that may even exert a more substantial impact on the risk of recurrence than the UICC stage.^43^ When combined, the risk score and the pT‐category showed even greater prognostic potential and were significantly associated with TTR for patients with pT3 tumors and pT4 tumors. Furthermore, the risk score was significantly associated with OS for patients with pT4 tumors. In addition, the risk score revealed that low‐risk patients in this cohort did not benefit from adjuvant chemotherapy, while high‐risk patients who received chemotherapy had a significantly worse TTR compared to those who did not. This suggests that the high‐risk patients who received adjuvant chemotherapy may have had chemotherapy‐resistant tumors or a more aggressive form of the disease, indicating the potential need for more targeted treatments for these individuals. For low‐risk patients, these findings suggest that some who were classified as low‐risk based on the RNA‐based risk score may have been misclassified according to the standard clinical criteria used to guide adjuvant chemotherapy treatment. Alternatively, it is possible that adjuvant chemotherapy improved the TTR in these patients, allowing them to achieve survival outcomes similar to those in the low‐risk group despite being classified as high‐risk by the standard clinical criteria.

Although our study shows great promise in risk‐stratifying patients with stage II colon cancer based solely on RNA transcripts that have shown prognostic value in other cohorts, our main limitation was the lack of another external validation cohort. However, this may be compensated by our multicenter patient cohort, which included an unbiased selection of all patients with stage II colon cancer across the entire region. Furthermore, to mitigate overfitting, only RNA transcripts significant in multivariate analysis were used, while we also utilized bootstrap methods to assess the stability of the model. Five Cancer Genome Atlas Program (TCGA) cohorts of colon cancer were screened as possible validation cohorts, but in all cohorts the expression of *SNORA2B* was only detected in very few or no patients. Furthermore, no TCGA cohorts utilized the latest UICC criteria (8th edition) for classification of tumors, and none contained information about participation in screening programs, and many patients had limited information concerning recurrence status, synchronous cancer, surgical margin status, and hereditary cancer. Thus, only a partial validation of the signature in a cross‐platform setting with patients who were not thoroughly characterized was possible, which we considered not to be meaningful.

## 5 CONCLUSION

In conclusion, we have developed a gene expression score, based on *ZNF697*, *CTSC*, *SNORA2B*, and *OXLD1*, that effectively stratifies patients with stage II colon cancer into low and high‐risk groups, facilitating the identification of patients who may benefit from adjuvant chemotherapy. These findings offer valuable insights for future investigations and clinical applications in the management of stage II colon cancer.

## AUTHOR CONTRIBUTIONS

**Ulrik Korsgaard:** Conceptualization; data curation; formal analysis; funding acquisition; validation; investigation; methodology; writing – original draft; project administration; writing – review and editing. **Maria P. Kristensen:** Data curation; investigation; writing – review and editing. **Juan L. García‐Rodríguez:** Investigation; methodology; writing – review and editing. **Sanne Kjær‐Frifeldt:** Data curation; writing – review and editing. **Jan Lindebjerg:** Supervision; writing – review and editing. **Torben F. Hansen:** Conceptualization; methodology; writing – review and editing. **Jørgen Kjems:** Conceptualization; funding acquisition; writing – review and editing; resources. **Henrik Hager:** Conceptualization; resources; formal analysis; supervision; validation; investigation; methodology; project administration; writing – review and editing. **Lasse S. Kristensen:** Conceptualization; methodology; investigation; validation; formal analysis; supervision; funding acquisition; project administration; resources; writing – review and editing.

## CONFLICT OF INTEREST STATEMENT

The authors have no conflicts of interest.

## ETHICS STATEMENT

The study was conducted in accordance with the Declaration of Helsinki. The study was approved by the Regional Committee on Health Research Ethics for Southern Denmark (Approval number: S‐20210009) with the requirement for patient consent to the specific analyses waived.

## Supporting information


**TABLE S1.** NanoString nCounter probes, their respective class of RNA, circAtlas_ID, circbase_ID, Gene symbol and target sequence.

**DATA S1:** Supporting Information.

## Data Availability

All pertinent data for this study is provided in text, figures, and in the supplementary files. Further information is available from the corresponding author upon request.
