# Supplementary material for: The prognostic potential of RNA in stage II colon cancer: Insights from a screened multicenter population‐based cohort study
Source: Int J Cancer. 2025 Jun 6;157(8):1746–55. doi: 10.1002/ijc.35507 (PMC12375848; doi:10.1002/ijc.35507)
Supplement: Supplementary file 2 — DATA S1: Supporting Information. [file IJC-157-1746-s002.pdf]

The Prognostic Potential of RNA in Stage II Colon Cancer: Insights from a Screened Multicenter Population-Based Cohort Study

Ulrik Korsgaard, Maria P. Kristensen, Juan L. García-Rodríguez, Sanne Kjær-Frifeldt, Jan Lindebjerg, Torben F. Hansen, Jørgen Kjems, Henrik Hager, Lasse S. Kristensen

Table of Contents

**Supplementary Table S1**	**Available in a separate file**
**Supplementary Table S2**	**2**
**Supplementary Table S3**	**3**
**Supplementary Table S4**	**5**
**Supplementary Table S5**	**6**
**Supplementary Figure S1**	**7**
**Supplementary Figure S2**	**8**
**Supplementary Figure S3**	**9**

Supplementary Table 1. NanoString nCounter probes, their respective class of RNA, circAtlas_ID, circbase_ID, Gene symbol and target sequence

Supplementary Table S2. Association of RNA transcripts from previously described gene signatures with 5-year recurrence.

Study	RNA	Univariate analysis		
**O'Connell MJ, et al.** [19]		OR	95 % CI	p-value
**Patients:** Colon, UICC stage II and stage III (n=1,851) **Method:** RT-qPCR	*BGN*	1.31	0.91-1.89	0.141
	FAP	1.40	0.92-2.17	0.125
	INHBA	**1.49**	**1.03-2.18**	**0.035**
	GADD45B	1.25	0.81-1.92	0.306
	MKI67	**0.49**	**0.31-0.78**	**0.002**
	C-MYC	0.67	0.39-1.11	0.125
	MYBL2	0.72	0.48-1.07	0.106
**Salazar R, et al.** [23]				
**Patients:** Colon and rectum, UICC stage I-IV (n=394) **Method:** Agendia customized whole-genome oligonucleotide high-density microarray	***CTSC***	**0.38**	**0.19-0.78**	**0.009**
	CYFIP2	0.96	0.57-1.59	0.871
	EDEM1	1.00	0.48-2.13	0.998
	HSD3B1	1.29	0.89-1.81	0.154
	IL2RA	**0.56**	**0.35-0.89**	**0.016**
	IL2RB	**0.54**	**0.35-0.82**	**0.004**
	LAMA3	1.40	0.90-2.16	0.131
	LIF	1.21	0.81-1.77	0.345
	MCTP1	0.80	0.47-1.34	0.394
	PIM3	1.17	0.58-2.35	0.650
	PLIN3	1.27	0.56-2.84	0.561
	PPARA	1.22	0.72-2.04	0.455
	PYROXD1	0.75	0.38-1.44	0.409
	SLC6A11 (ULoD)		-	
	THNSL2	0.90	0.68-1.20	0.447
	ZBED4	1.09	0.43-2.73	0.849
	ZNF697	**2.66**	**1.56-4.55**	**<0.001**
	CA4388O2 (PDNP)		-	
**Huang X, et al.** [28]				
**Patients:** Colon and rectum, UICC stage I-IV (n=480) **Method:** Total RNA-sequencing from TCGA database.	CLCA4-AS1	1.07	0.61-1.75	0.8
	LINC01485 (ULoD)		-	
	LINC02257	1.10	0.81-1.49	0.5
	RBAKDN	0.85	0.51-1.38	0.5
	Lnc-GLDN-1 (ULoD)		-	
	Lnc-SLCO5A1-2	1.36	0.84-2.15	0.2
**Gao M, et al.** [27]				
**Patients:** Colon, UICC stage I-IV (n=411) **Method:** Total RNA-sequencing data from the TCGA database.	PGGT1B-AS (ULoD)		-	
	EVX1-AS	1.27	0.90-1.76	0.2
	LINC02474	0.94	0.70-1.23	0.7
	TMEM220-AS1	0.98	0.62-1.48	0.9
	ZNF667-AS1	1.50	0.85-2.58	0.1
**Zheng Y, et al.** [25]				
**Patients:** Colon and rectum, UICC stage I-IV (n=1,139) **Method:** Total RNA-sequencing data from the TCGA and GEO database	*CCND1*	0.98	0.49-1.96	0.96
	EDAR	**1.27**	**1.02-1.61**	**0.04**
	FUT1	1.05	0.64-1.66	0.83
	PPAT	**0.51**	**0.27-0.97**	**0.04**
	CCNB1	**0.59**	**0.36-0.97**	**0.04**
	CDC6	**0.54**	**0.35-0.87**	**0.01**
	GPD1L	0.81	0.44-1.50	0.50
	MAD2L1	0.74	0.49-1.12	0.15
	MMP1	0.94	0.76-1.16	0.55
	SPP1	**1.30**	**1.01-1.69**	**0.04**
	TKT	**0.44**	**0.20-0.95**	**0.04**
**Ju HQ, et al.** [29]				
**Patients:** Colon, UICC stage II-III (n=667) **Method:** Total RNA sequencing for discovery and RT-qPCR for validation	hsa_circ_0122319	1.21	0.70-2.07	0.5
	hsa_circ_0008039	0.71	0.44-1.11	0.1
	hsa_circ_0087391 (ULoD)		-	
	hsa_circ_0079480 (PDNP)		-	

ULoD = under limit of detection. PDNP = probe design not possible. Significant RNAs are marked in bold.

Supplementary Table S3. Univariate logistic regression for time to recurrence (TTR) of RNAs from our previous RNA-sequencing study.

Target probe	Class	p-value	OR	OR lower	OR upper
**SNORA2B**	**snoRNA**	**0,002190612**	**2,309011091**	**1,351707571-**	**3,944294117**
**OXLD1**	**mRNA**	**0,006607541**	**0,351986365**	**0,165684837**	**0,747771512**
**MAPRE3**	**mRNA**	**0,007072187**	**2,095376156**	**1,223163903**	**3,589544478**
**SNHG1**	**lncRNA**	**0,018957281**	**0,380862033**	**0,170049582**	**0,853021138**
**IFI35**	**mRNA**	**0,020150769**	**0,519900094**	**0,299426387**	**0,902713051**
**MSLN**	**mRNA**	**0,037333683**	**1,192835844**	**1,010397104**	**1,408215983**
**circ_00038_TULP4**	**circRNA**	**0,046863846**	**1,850750587**	**1,008574065**	**3,396158846**
**C6ORF15**	**mRNA**	**0,04717161**	**1,339437242**	**1,003657243**	**1,787554603**
circ_00198_GCN1	circRNA	0,060101436	0,53888772	0,282867706	1,026628237
EIF2AK2	mRNA	0,073729842	0,502158201	0,236026862	1,06836509
TGFBR1	mRNA	0,087508456	1,82441517	0,915377007	3,636196549
TMEM159	mRNA	0,089238375	2,013092113	0,898287739	4,511405068
RASA2	mRNA	0,090676146	2,003396439	0,895727976	4,480821631
circ_00067_RBM33	circRNA	0,105780094	1,746918682	0,888580614	3,434381565
PIGR	mRNA	0,10645698	0,922352987	0,836139102	1,017456342
VTRNA1-3	vtRNA	0,110890875	1,521525098	0,908195134	2,549054202
SNORA53	snoRNA	0,115709685	1,587774102	0,892506702	2,824658452
circ_FNDC3B_1.1	circRNA	0,134588571	1,691356355	0,849700578	3,366699275
GSK3A	mRNA	0,145671201	0,512729606	0,208492498	1,260916586
circ_00311_PSME3IP1	circRNA	0,149257694	0,635074407	0,342652251	1,177051957
AFAP1.AS1	lncRNA	0,207699838	0,839566196	0,639605869	1,102040228
UBXN2A	mRNA	0,21342409	0,534752334	0,199488872	1,433463707
SFRP2	mRNA	0,216917956	1,202251441	0,897464688	1,610546406
circ_00082_NFAT5	circRNA	0,21877694	0,624243145	0,294578016	1,322839732
circ_ZNF91.1	circRNA	0,238515265	0,788825151	0,531715502	1,170259504
circ_RBM33	circRNA	0,244671773	1,446443434	0,776678367	2,693777367
HNF4A	mRNA	0,266819297	1,201392962	0,869031139	1,660866895
SLC25A5-AS1	lncRNA	0,282682744	1,295561839	0,807775524	2,077904601
LASP1	mRNA	0,293426164	1,605601481	0,663773233	3,883790407
SNHG5	lncRNA	0,314409725	0,859265297	0,639426569	1,154685912
circ_ciRS-7	circRNA	0,315342584	1,191851773	0,846127201	1,67883818
VTRNA2-1	vtRNA	0,352155554	0,854804077	0,614243367	1,189577372
PDXP	mRNA	0,378160743	0,73972291	0,378340185	1,446290944
TEP1	mRNA	0,414741872	1,252610864	0,729034601	2,152207829
circ_00014_MALAT1	circRNA	0,417192681	1,17910212	0,79194592	1,755526196
circ_00050_PILRB	circRNA	0,429273702	1,349122344	0,642060374	2,834828581
MRPL30	mRNA	0,431864417	0,79031322	0,439494938	1,421165368
HSD17B1-AS1	lncRNA	0,453491929	1,308310586	0,647965456	2,641617042
KIAA1671-AS1	lnRNA	0,464306095	1,187640993	0,749291102	1,882434111
CARD8-AS1	lncRNA	0,466643003	0,796833297	0,432320385	1,46868694
EIF4EBP3	mRNA	0,470953348	1,226648265	0,703914534	2,137569111

Supplementary Table S3 continued

Target probe	Class	p-value	OR	OR lower	OR upper
ZC3H7B	mRNA	0,476972792	1,381929505	0,566651704	3,370199265
VTRNA1-2	vtRNA	0,48020687	1,109837863	0,831011012	1,482218723
ADNP-AS1	lncRNA	0,497782769	1,192529516	0,716813674	1,983955798
circ_CDYL.1	circRNA	0,508663234	0,773520989	0,361189782	1,656566019
circ_00046_FAM13B	circRNA	0,513901445	1,218599269	0,673073562	2,206273228
circ_00079_MALAT1	circRNA	0,520326745	1,182532668	0,709271875	1,971576146
circ_ZKSCAN1.1	circRNA	0,524272758	1,213110501	0,669357436	2,198581815
HHLA3	mRNA	0,527076904	1,202140516	0,67949045	2,126802251
circ_HIPK3.1	circRNA	0,53346029	1,229238345	0,641976245	2,35371156
AKAP12	mRNA	0,568406927	1,099376104	0,793893835	1,522404841
circ_00071_ATXN1	circRNA	0,57503404	1,187126144	0,651727953	2,16235697
ATP1A1-AS1	lncRNA	0,577817483	0,829202339	0,428771565	1,603596356
circ_00009_CCDC9	circRNA	0,59942298	0,861279135	0,493316091	1,503704748
circ_ZNF609.1	circRNA	0,603330392	0,83232919	0,416536703	1,663171277
circ_00129_RBM39	circRNA	0,662625753	0,899854463	0,560142895	1,445591942
WBP2	mRNA	0,667322523	1,234143508	0,472875577	3,220953403
PRSS22	mRNA	0,672063668	1,076392289	0,765502271	1,513542682
TSHZ1	mRNA	0,713108739	1,145847423	0,554558864	2,367586927
ZNF710-AS1	lncRNA	0,725192142	0,913228294	0,550538226	1,514855605
circ_00098_MALAT1	circRNA	0,728815529	0,952595826	0,723895305	1,25354979
Lnc-USP34-1	lncRNA	0,729531835	0,901192416	0,499676594	1,625346835
GAS5	lncRNA	0,730881942	0,907423008	0,521668928	1,578427372
OLFM4	mRNA	0,732442162	1,018420485	0,917236929	1,130765945
PRKACA	mRNA	0,765428056	1,153292808	0,451909414	2,943254244
LOC101929719	lncRNA	0,773471838	0,924210998	0,5403768	1,580685864
circ_00328_MVP	circRNA	0,811359265	1,083440861	0,561031667	2,092295622
MFAP1	mRNA	0,814692571	0,904944095	0,392530272	2,086269197
circ_00061_SUPT5H	circRNA	0,85604246	1,054800748	0,592709126	1,877151151
PPP1R12A	mRNA	0,878689729	1,079754322	0,403079999	2,892402002
circ_00006_MALAT1	circRNA	0,920005302	0,980385597	0,666034463	1,44310238
GTF2I	mRNA	0,921202508	1,044869274	0,437897003	2,493170296
WDR70	mRNA	0,930573013	1,032406765	0,503800325	2,115647166
circ_00303_RERE	circRNA	0,933524468	1,028780723	0,528161243	2,003914126
PPP1R9B	mRNA	0,942803312	0,963179694	0,345643216	2,684025261
SLC35A4	mRNA	0,946366076	1,016814048	0,625541854	1,652824352
MAP3K11	mRNA	0,965497235	0,984232101	0,47900822	2,022330281
FAM222B	mRNA	0,981595901	1,008091168	0,508325463	1,99920696

Supplementary Table S4. Receiver operating characteristics (ROC) analysis of RNAs associated with time to recurrence (TTR).

RNA	AUC
**ZNF697**	**0,675005935**
**MAPRE3**	**0,638117284**
**CDC6**	**0,63384378**
**IL2RB**	**0,632122507**
**MKI67**	**0,629036087**
**SNORA2B**	**0,620667142**
**IFI35**	**0,619420703**
**CTSC**	**0,618649098**
**OXLD1**	**0,61811491**
**SNHG1**	**0,609449193**
IL2RA	0,605413105
EDAR	0,603751187
MSLN	0,598438984
INHBA	0,597875119
CCNB1	0,596747388
SPP1	0,594313865
C6ORF15	0,585885565
TKT	0,581730769
PPAT	0,575498575
circ_00038_TULP4	0,575261159

Supplementary Table S5. Association of the risk-score with clinicopathological criteria.

Variable	Overall, N=471[1]	High-risk, N=89[1]	Low-risk, N=382[1]	p-value[2]
**Recurrence**	39 (8.3)	23 (26)	16 (4.2)	**<0.001**
**Age**	73 (67, 80)	72 (67, 79)	74 (67, 80)	0.2
**Gender**				0.13
Female	252 (54)	54 (61)	198 (52)	
Male	219 (56)	35 (39)	184 (48)	
**Histological Subtype**				0.9
Adenocarcinoma	404 (86)	76 (85)	328 (86)	
Medullary carcinoma	3 (0.6)	0 (0)	3 (0.8)	
Mucinous adenocarcioma	63 (13)	13 (15)	50 (13)	
Undifferentiated carcinoma	1 (0.2)	0 (0)	1 (0.3)	
**Tumor Location**				**0.015**
Cecum	79 (17)	8 (9)	71 (19)	
Ascending Colon	83 (18)	10 (11)	73 (19)	
Right Colic Flexure	36 (7.6)	4 (4.5)	32 (8.4)	
Transverse Colon	43 (9.1)	8 (9)	35 (9.2)	
Left Colic Flexure	31 (6.6)	6 (6.7)	25 (6.5)	
Descending Colon	34 (7.2)	9 (10)	25 (6.5)	
Sigmoid Colon	165 (35)	44 (49)	121 (32)	
**Tumor Differentiation**				0.3
Moderate	432 (92)	84 (94)	349 (91)	
Poor	38 (8.1)	5 (5.6)	33 (8.6)	
**Mismatch Repair Status**				**0.013**
dMMR	117 (25)	13 (15)	104 (27)	
pMMR	354 (75)	76 (85)	278 (73)	
**T-category**				**0.016**
pT3	420 (89)	73 (82)	347 (91)	
pT4	51 (11)	16 (18)	35 (9.2)	
**Lymph nodes**				0.7
<12	9 (1.9)	2 (2.2)	7 (1.8)	
>12	462 (98)	87 (98)	375 (98)	
**Acute intervention**				**<0.001**
Yes	46 (9.8)	19 (21)	27 (7.1)	
No	425 (90)	70 (79)	355 (93)	
**Anastomosis leak**				0.5
Yes	15 (3.2)	4 (4.5)	11 (2.9)	
No	456 (97)	85 (96)	371 (97)	
**Venous invasion**				0.053
Yes	145 (31)	35 (39)	110 (29)	
No	326 (69)	54 (61)	272 (71)	
**Lymphatic invasion**				>0.9
Yes	25 (5.3)	4 (4.5)	21 (5.5)	
No	446 (95)	85 (96)	361 (95)	
**Perineural invasion**				**0.002**
Yes	59 (13)	20 (22)	39 (10)	
No	412 (87)	69 (78)	343 (90)	

[1]n (%); Median (IQR). [2]Pearson's Chi-squared test; Wilcoxon rank sum test; Fisher's exact test.

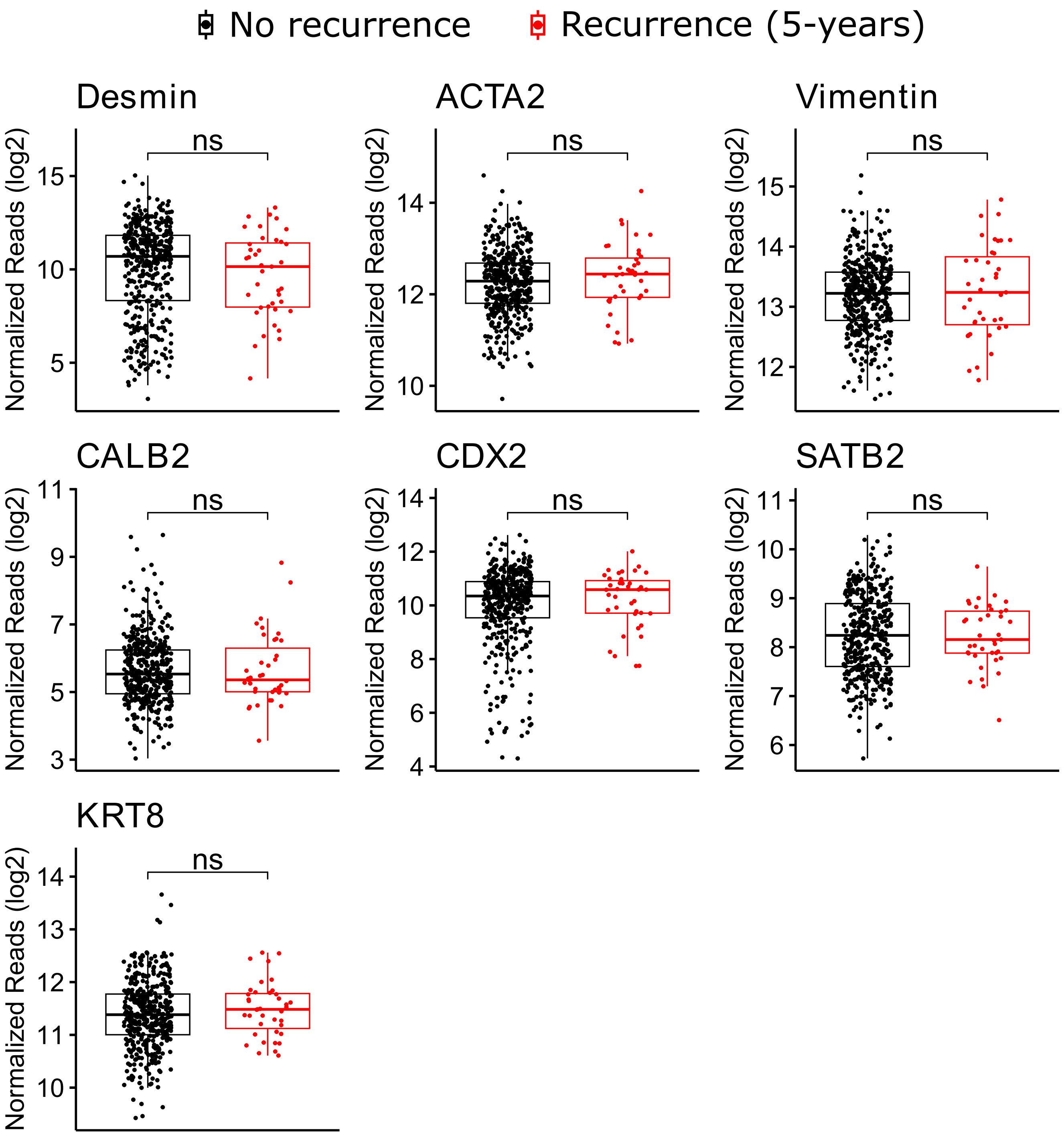

Supplementary Figure S1: Expression of tissue specific mRNAs between patients with recurrence and without recurrence. Boxplots with normalized log2 transformed nCounter reads on y-axis, patients without recurrence in black and patients with recurrence in red.

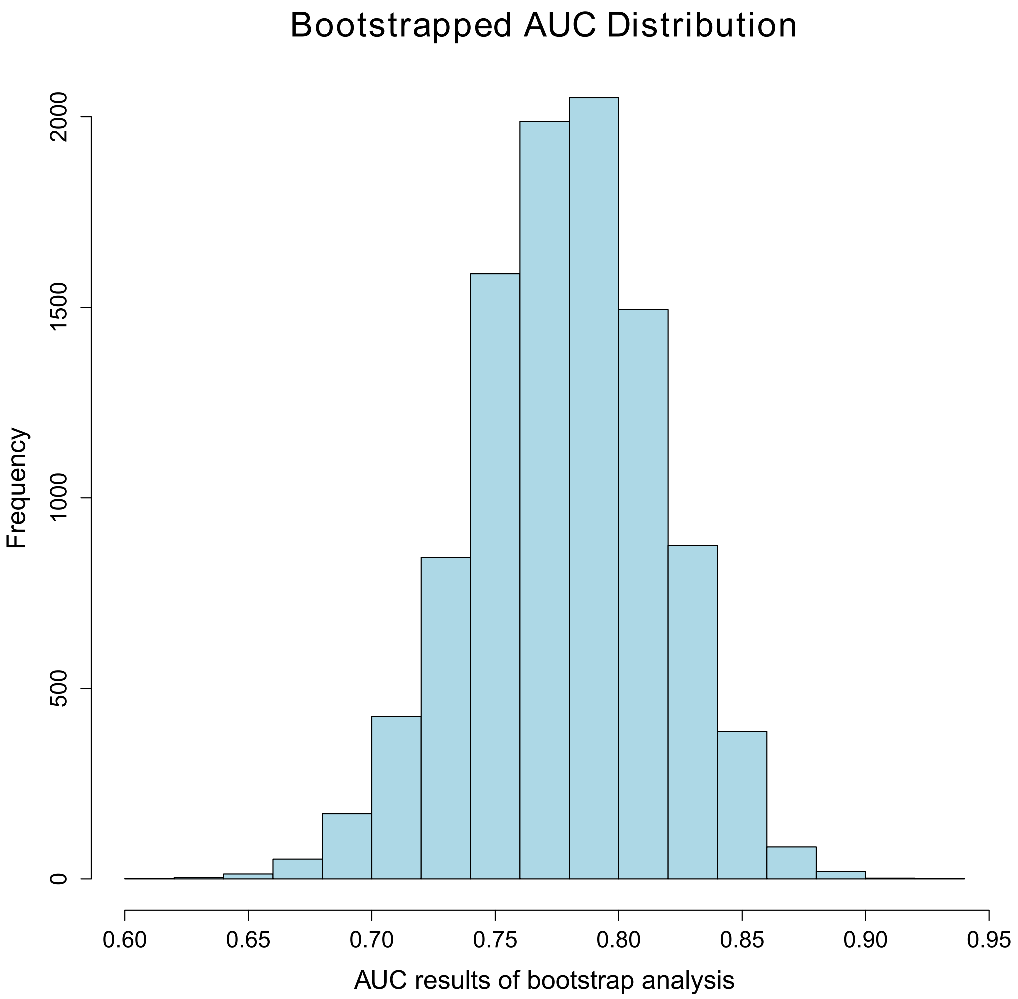

Supplementary Figure S2: Bootstrap analysis. Bootstrap analysis performed with 10,000 iterations to assess the stability of the prognostic model. Frequency for which a particular area under the curve (AUC) was obtained in bootstrap analysis (y-axis) and AUC obtained (x-axis).

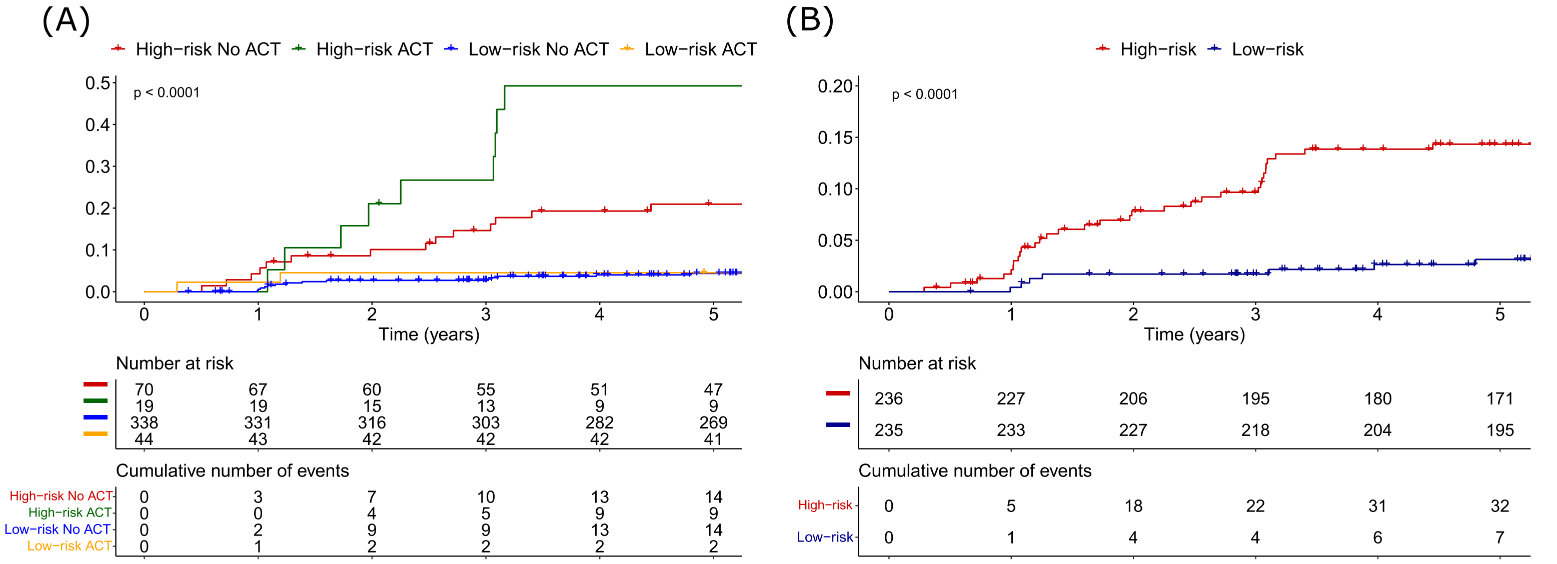

Supplementary Figure S3: Kaplan Meier analysis for time to recurrence (TTR). (A) Kaplan Meier plot showing a stratified analysis on how low-risk and high-risk patients risk of recurrence are affected according to chemotherapy treatment. (B) Kaplan Meier analysis for TTR using the risk-score, with patients divided into high-risk and low-risk groups based on whether their risk score is above (high-risk) or below (low-risk) the median of the risk-score distribution.
